# Supported Standing and Supported Stepping Devices for Children with Non-Ambulant Cerebral Palsy: An Interdependence and F-Words Focus

**DOI:** 10.3390/ijerph21060669

**Published:** 2024-05-23

**Authors:** Ginny S. Paleg, Sian A. Williams, Roslyn W. Livingstone

**Affiliations:** 1 Independent Researcher, Silver Spring, MD 20901, USA; 2 School of Allied Health, Curtin University, Perth, WA 6009, Australia; sian.williams@curtin.edu.au; 3 Liggins Institute, University of Auckland, Auckland 1023, New Zealand; 4 Occupational Science and Occupational Therapy, Faculty of Medicine, University of British Columbia, Vancouver, BC V6T 2B5, Canada; roslyn.livingstone@ubc.ca

**Keywords:** standing frame, stander, gait trainer, support walker, assistive devices

## Abstract

Children functioning at Gross Motor Function Classification System (GMFCS) levels IV–V cannot maintain an aligned standing position or take steps without support. Upright positioning and mobility devices have psycho-social significance for these children and their families, enhancing use of vision, communication, functioning and emotional well-being. Standers and supported stepping devices facilitate opportunities for biomechanical loading, potentially helping to build and maintain muscle and bone integrity, and they promote physical development. However, families are often required to choose between these two devices for their young child. This study aims to synthesize evidence for use and benefits of both supported standing and stepping devices through the lens of two contemporary theoretical frameworks to support clinical reasoning and implementation. The F-words for childhood development (functioning, family, fitness, fun, friends, future) and the interdependence-Human Activity Assistive Technology (iHAAT) models were combined to illustrate the complex interactions between the child, family, caregivers, peers and contextual factors when implementing standing and stepping devices with children at GMFCS levels IV and V. Supported standing and stepping devices provide complementary benefits, and both may be necessary starting at 9–15 months. We propose they both be included ON-Time, along with other age-appropriate positioning and mobility devices, to promote more equitable developmental opportunities for children with non-ambulant cerebral palsy.

## 1. Introduction

The body of evidence regarding therapeutic interventions for cerebral palsy (CP) is quite large, but the vast majority focuses on children who are ambulant (Gross Motor Function Classification System [1] (GMFCS) levels I, II and III) [2]. CP-specific interventions can improve outcomes in body structure and function (BSF), activity and participation. Moreover, the importance of early and age-appropriate interventions has been emphasized [3]. Interventions should be child-active and directed, with practice of activities taking place within meaningful family or school routines [3]. However, children with different levels of impairment, topography, movement disorders or concurrent morbidities may respond differently [4], and there is growing concern that not all children with CP are afforded the same opportunities for practice or evidence-based interventions [5].

Children classified at GMFCS levels IV and V make up between 24.0 and 32.5% [6,7,8] of the CP population (with higher percentages in lower-resourced settings) and are considered non-ambulant. These children use wheeled mobility devices with physical or powered assistance in most settings [1], and they are more likely to experience secondary musculoskeletal impairments, pain and functional limitations [9]. Children with non-ambulant CP never develop efficient quadruped crawling or walking and require assistive devices for upright positioning and mobility [1,10].

Children at the greatest risk of being non-ambulant can now be identified by 3–5 months of age, helping target management and early intervention strategies appropriately. A General Movement Assessment Motor Optimality Score (GMA-MOS) below 8 at 2–5 months of corrected age and a Hammersmith Infant Neurological Examination (HINE) score below 40 between 3 and 24 months of age are the current cut-off scores assisting the identification of children at the highest risk of being classified as GMFCS levels IV or V [11,12,13,14]. While a few children functioning at GMFCS level IV may be able to stand briefly without support, most classified at GMFCS levels IV/V use devices known as standers, standing frames, standing shells or braces [15]. Children functioning at GMFCS levels IV/V may use supported stepping devices (also referred to as body support walkers or gait trainers) to move around at home or school [16,17].

Mobility is a human right and, to be ‘ON-Time’ or age-appropriate developmentally, multiple positioning and mobility modes are needed starting within the first year of life [18]. In comparison to prone mobility (crawling), our visual field is significantly expanded in upright positions [19], and the use of an infant walker has been shown to facilitate spatial search performance [20]. When supported in an upright position, the expanded visual field increases eye contact with peers and caregivers, potentially enhancing participation and increasing equity in age-appropriate developmental experiences. 

Supported standing and stepping devices can promote functioning, fitness and overall health, emotional development, inclusion and participation in home and school settings for children with non-ambulant CP [21,22]. Development of independent standing or stepping without the devices is not an expectation, and power mobility is the focus for efficient functional mobility for children at GMFCS levels IV/V, especially in community settings [3,22,23]. 

From around the age of 12–15 months, muscle volumes of children with CP appear to ‘separate’ from those of typically developing children [24,25]. This is the age when children are typically pulling to stand, playing in a standing position and exploring their environment by stepping. In children with CP, an altered and reduced biomechanical loading of muscle (related to patterns of reduced standing, walking and physical activity) is proposed as a contributing factor [26,27,28], with early and consistent activity being the antidote. However, increasing physical activity and decreasing sedentary behavior is challenging, and few recommendations target, or are appropriate for, children with non-ambulant CP [29]. 

The morphological and structural changes reported in CP muscle in early childhood may increase by up to 43% by adolescence [30,31,32], whilst increases in inter- [33] and intramuscular [34] adiposity [35], excess connective tissue [36,37], and changes in fiber type distribution and size are also evident. There are strong indications that not only do these changes in the muscle increase in individuals classified by higher GMFCS levels [32,38] but they are also hypothesized to continue to decline with age, with additional degenerative complications from chronic stress (resulting from overactive and co-contracting muscles, and movement inefficiencies) as well as from reduced physical activity. 

Individuals with CP face an earlier and accelerated age-related functional decline and increased risk to cardiometabolic health [39]. Combined with low bone mineral density (BMD) [40], adults with CP are at an increased risk of “osteosarcopenia”, the co-existence of both osteoporosis and sarcopenia, which increases the risk of fragility fractures, causing pain and leading to greater morbidity and mortality as well as higher socioeconomic costs [41]. The morphological and structural properties of a muscle not only influence its ability to generate strength and support function but they are also important for the provision of metabolically active lean tissue available for glucose storage and metabolism [39], underscoring the important role of muscle for cardiometabolic health.

A scoping review of supported standing device use with children and young adults with non-ambulant CP [21] summarized and evaluated results from 16 systematic reviews, six clinical guidelines and 37 primary studies. Data from 1101 individuals from 7.2 months to 25 years of age were included in experimental, descriptive and qualitative studies. The findings of the review highlighted that evidence for the maintenance of BMD and prevention of contractures was well supported by experimental studies, with other outcomes (such as hip stability, bowel function, spasticity management, activities of daily living and gross and fine motor function) being currently supported primarily by quasi-experimental or descriptive evidence. Across the literature, use of supported standing devices is described as being influenced by the individual, the device and other factors in the physical, social and attitudinal environment. 

In a second scoping review, this time focused on supported stepping device use with children and adults with non-ambulant CP. Ref. [22] summarized and critiqued results from eight systematic or scoping reviews, two clinical guidelines and 59 primary studies. Fifteen of the included 59 primary studies were experimental designs and four were qualitative or mixed-methods, with the remainder being primarily observational or descriptive studies culminating in data from 705 individuals (ages 9 months to 47.7 years) and 632 therapists. This review concluded that despite limited experimental evidence, included data supports a positive impact of supported stepping devices on stepping and other physical abilities, as well as on individual self-esteem, independence and autonomy. Lived-experience data similarly confirm the influence of the individual’s profile, the type of device and other environmental factors. 

Although these recent scoping reviews examined what is known about the use of supported standing [21] and supported stepping [22] devices, no studies have compared or contrasted the evidence, outcomes and benefits of these two types of devices, with support for clinical reasoning lacking. Both reviews confirmed the influence of environmental factors, particularly the device (assistive technology) and the influence of others (social and attitudinal environment) in the use of supported standing and stepping devices. Despite the importance of using theory to guide research, assist in data interpretation and evaluate intervention choices, the use of theoretical frameworks in assistive technology (AT) outcomes research is extremely limited [42]. Dissemination and implementation science is a growing field, aiming to help bridge the gap between what is known and what is done in practice, as well as to increase equitable access to evidence-based interventions [43]. Although systematic or scoping reviews can summarize and evaluate the strength of evidence for specific interventions, a clinically focused synthesis is needed to support decision making and the implementation of these complementary interventions in practice. 

As summarized in the two scoping reviews, supported standing and stepping devices afford age-appropriate physical activity, developmental experiences and opportunities. The use of both these devices may help to increase and vary opportunities for position change, weight bearing and active movement. In addition, they may help to reduce sedentary behavior and facilitate participation and engagement in different activities throughout the day [21,22,44]. Unfortunately, in many practice settings, funders do not allow the provision of both standing and stepping devices ‘ON-Time’ (starting at 9–12 months) and require therapists and families to choose one or the other. Synthesizing and comparing the evidence (as established within the two scoping reviews) in relation to contemporary theoretical frameworks may help to support clinical reasoning and practice implementation. 

The purpose of this article is to frame and synthesize the best available evidence supporting the use of standing and stepping devices with children classified as non-ambulant CP through the lens of two contemporary theoretical frameworks: the F-words (functioning, family, fitness, fun, friends and future) for childhood development [45] and the interdependence-Human Activity Assistive Technology (iHAAT) model [42,46]. Therapists will be challenged to consider that both standing and stepping devices may be necessary to provide equitable access to positioning and mobility opportunities ‘ON-Time’ for young children with non-ambulant CP.

## 2. Materials and Methods

The two scoping reviews of the entire body of evidence related to (1) the use of supported standing devices [21] and (2) supported stepping devices [22] with those classified at GMFCS levels IV and V provide comprehensive and up-to-date evidence summaries. Readers are referred to the original publications for details of the search strategy, methods and results. In this study, results and findings from both reviews were further classified, compared and contrasted in relation to two theoretical frameworks, the F-words and the iHAAT. 

Lived-experience data were analyzed in relation to the F-words within the published manuscript [22] by both authors of the review on supported stepping and by all authors of the review on supported standing [21] for a conference presentation [47]. Qualitative and quantitative data from both reviews were later compared and contrasted according to the F-words by two authors (GSP and RWL) for another conference presentation [48]. Analysis of data from both reviews in relation to the iHAAT framework was conducted specifically within this study. In each case, authors analyzed data independently and agreed on the findings and synthesis through discussions. 

### 2.1. F-Words for Child Development

**Figure 1:**
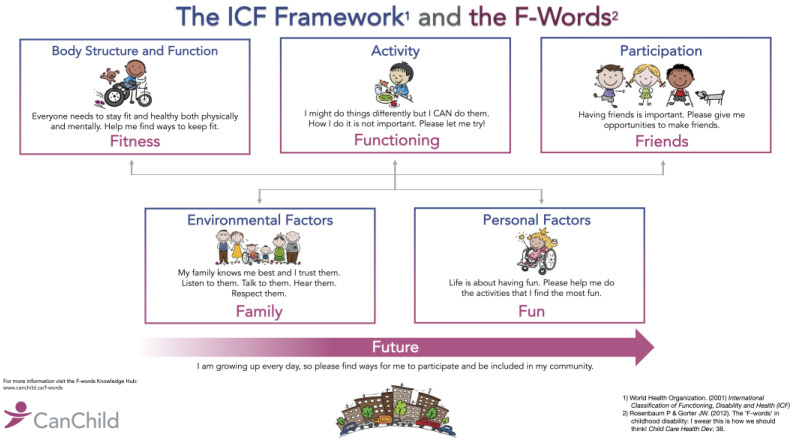
ICF-F-words figure [49,50]. Used with permission of CanChild’s F-words Knowledge Hub: www.canchild.ca/f-words, accessed on 19 May 2024. Copyright 2023, McMaster University.

The F-words for childhood development [45] (see Figure 1) enhance our understanding of the International Classification of Functioning, Disability and Health (ICF) [49] and are a child- and family-friendly way of incorporating ICF concepts into everyday life. The words used are meaningful to children and families and facilitate knowledge translation. Functioning combines aspects of activity and participation; fitness relates to BSF; friends is related to participation; fun is a combination of personal factors and participation; family is the most important environmental factor for children; and future, while not included in the ICF, includes interventions designed to promote future health and development or to prevent known harms [45,50,51].

Under the ICF, assistive devices, such as standing and stepping devices, fall under environmental modifications, which translates to family within the F-words framework [45,51]. However, assistive technology or devices (AT) provide benefits across all F-words [50], and intervention methods may be more clearly linked to F-words based on the goals set or outcomes measured [52]. For example, if a supported standing or stepping device is being used to, e.g., maintain BMD or prevent contractures, it may be assigned to fitness; if it is being used to increase participation in age-appropriate activities with others, it may be assigned to friends, and so on. By associating intervention ingredients with F-words, they can be matched to child and family goals [50,51,52]. 

Interventions focused solely on fitness or BSF rarely result in improvements ‘upstream’ in the other F-words or ICF domains [2]. However, by addressing child and family goals and priorities for participation and engagement in meaningful activities, the resulting practice intensity may lead to fitness benefits [3]. Children and families are more likely to use assistive devices when they increase participation and facilitate the achievement of age-appropriate goals [50]. A strength of the F-words is the focus on accomplishment and joy in the moment, rather than a mythical ‘fix’ in the future. 

### 2.2. Interdependence-Human Activity Assistive Technology (iHAAT) Framework

The Human Activity Assistive Technology (HAAT) model is the most longstanding and widely accepted assistive technology framework, illustrating the dynamic interaction between the human (abilities, needs and roles), the activity and the assistive technology or device (AT) within the physical, social and attitudinal context or environment [53]. The activity component includes both activity and participation according to ICF language, and it includes functioning, friends and aspects of fun according to the F-words. Traditionally, assistive devices are prescribed to compensate for lack of ability and promote independence [54]. However, supported standing and stepping devices require caregiver support for successful implementation with children functioning at GMFCS levels IV/V, and all who interact with the child and the device influence their prescription and use [21,22]. 

There is a continuum from independence to interdependence, and quality of life is influenced by the social circle around all individuals [55]. An interdependence frame emphasizes the relationships between all people interacting with the assistive device and the environment [54]. Recently, the interdependence frame has been merged with the HAAT model to form the interdependence-Human Activity Assistive Technology (iHAAT) model [42,46]. 

**Figure 2:**
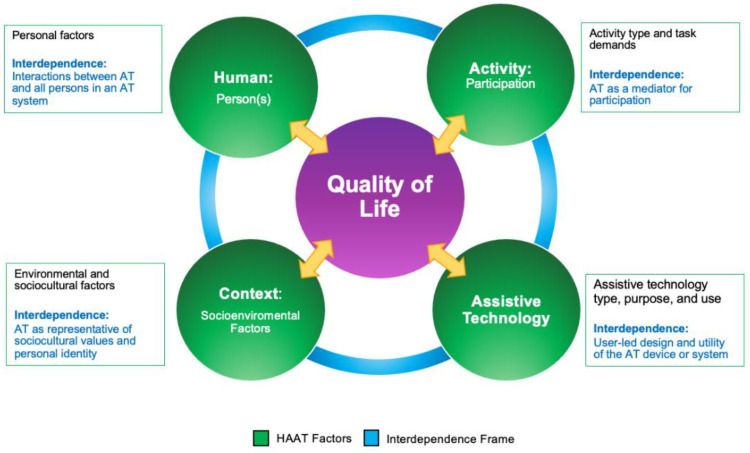
iHAAT framework. Used with permission, copyright 2022, Fani Lee [46].

The iHAAT conceptual framework illustrates how AT may afford increasing participation and engagement in meaningful activities, both for the individual and for others in their circle. An interdependence frame emphasizes “being and doing together” [54] (p. 169). The human(s) are interdependent with the AT and each other, appropriately matched AT promotes participation in desired activities for the human and others within their physical and social context, and all domains of the iHAAT interact to influence quality of life [42,46]. Access (to activities and the environment) and participation may be increased, and dependence may be reduced, without the expectation of complete physical or cognitive independence [54]. For children with non-ambulant CP, the goal may be to increase engagement and autonomy, rather than the child being able to complete tasks without assistance. See Figure 2.

## 3. Results

Evidence (overall conclusions as well as included study, guideline and review results) from the two scoping reviews [21,22] is compared and contrasted—first in relation to the F-words and then in relation to the iHAAT framework. The reviews on supported standing and stepping are cited where the results are summarized from a number of included studies or where overall findings/review conclusions are reported. Where statements, recommendations or conclusions are specific to the included individual studies, guidelines or reviews, the primary source is cited. Study design/evidence type and study quality are summarized according to the Mixed-Methods Appraisal Tool (MMAT) [56], as rated and reported in the original scoping reviews [21,22]. 

### 3.1. F-Words for Child Development

#### 3.1.1. Functioning

Increased mobility (including stepping, walking speed or distance) was the most highly reported outcome (588/705 participants) for supported stepping interventions [22]. Parents also described stepping as more effective in promoting indoor exploration than self-propelling a manual wheelchair [57]. Using a supported stepping device is considered ‘walking’ for many individuals with CP and their families. In personal communication, Peter Rosenbaum has shared that “for most families—and an increasing number of service providers—it is the achievement of ‘functioning’ that is important, not how it is done”. When a child has significantly impaired motor development, there is a shift in goals to a ‘new normal’ that may look very different from ‘typical’ development but is designed to promote development. Just because a child accomplishes their goal differently, does not lessen the value [58].

Increased participation in standing transfers and activities of daily living (ADL) have been measured following both standing [59] and stepping interventions [22]. Increased use of arms and hands for play and self-feeding was the second most highly reported outcome in the review on supported stepping, although only for studies involving hands-free supported stepping devices [22]. Increased attention, use of vision and improved communication outcomes were also widely reported [22]. Improved access to communication devices, increased hand use, self-feeding abilities and independence in play were also reported following the use of standing devices [21]. Improved head and trunk control was highly reported in the review on supported stepping [22], while increased gross motor function was reported following the use of standing devices in a case study [60] and a small case series [61]. 

#### 3.1.2. Family

Both family and other adults were considered under this F-word (family) [51,52], and both reviews addressed the impact of the social and attitudinal environment in relation to the use of standing and stepping devices [21,22]. Parents were ‘mostly’ or ‘very’ satisfied with supported stepping devices [16,57,62,63,64], apart from difficulties with transfers [57], particularly with increasing age of the child [17,65]. Easing caregiving and reducing burden of care was reported as a benefit for both devices [57,63,66,67]. 

#### 3.1.3. Fitness

The strongest evidence (positive evidence from experimental studies at low risk of bias) supports the use of supported standing to assist with the maintenance of BMD and the prevention of contractures [21]. Time weight bearing may be a critical factor for the maintenance of BMD in either standing [68,69,70] or stepping devices [17], with increased time associated with increased density. Improved bowel function has been measured in a qualitative [71] study as well as a feasibility study comparing participants using stepping devices to participants undergoing a standing program [17]. Two case studies [65,66] and anecdotal reports also suggest a positive impact on bowel function from upright positioning in supported standing. 

Supported standing is not necessarily a passive activity for children with non-ambulant CP. It may increase heart rate [72] and energy expenditure [73], and it is recommended as part of a 24 h activity guide to reduce sedentary behavior [74]. However, the potential for mobility and overground training would argue that supported stepping devices are likely to have a greater impact on physical fitness [22], and positive energy expenditure has been measured in response to moderate to vigorous intensity power training in a supported stepping device [75]. Clinical guidelines recommend overground supported stepping for bone health [76] and for increasing physical fitness in children with non-ambulant CP [3]. 

Evidence for the impact of supported standing on hip health is positive, although lower quality, and 1 h weight bearing daily is the most common dosage recommendation [21]. The influence of hip abduction during weight bearing is still unclear, although at least 10–15 degrees of abduction is recommended [42], due to possible negative effects of weight bearing with feet together [21]. A pilot randomized controlled trial found improved hip stability from increased time (up to 1 h) in supported standing despite difficulties maintaining adherence in this complex population [77]. No studies have examined the influence of supported stepping device use on hip stability, although expert opinion evidence supports this outcome [22]. Moreover, the opportunity for actively shifting one’s own weight may benefit bone and joint health [42].

Although no studies have examined the impact of supported standing or stepping on muscle development, decreasing sedentary behavior, increasing activity and facilitating opportunity for biomechanical loading of the muscle and bone through both supported standing and stepping devices could be an effective strategy to build and maintain muscle and bone integrity. Muscle hypertrophy (i.e., increasing the size of the muscle) is typically targeted by means of resistant training (where the muscles are working against a force or “load”); however, loading of the muscle for hypertrophic gains (particularly in groups with low levels of activity) could potentially be met with the person’s own body weight through standing or stepping.

#### 3.1.4. Fun

Stepping devices can facilitate joy in experiencing independent movement, particularly for children who have limited movement abilities [22]. Increased happiness, autonomy and self-efficacy were also frequently mentioned with increased engagement in typical childhood experiences, including being naughty, sneaking up on others and running away [57,71,78]. Experiences of fun in supported standing were mixed, and a strong theme that emerged in the standing review was that children need choice in where and when to stand. In order to be effective, standing programs must be integrated into age-appropriate and meaningful activities with others that facilitate peer interaction and participation [21].

#### 3.1.5. Friends

Upright positioning has psycho-social benefits that impact a child’s self-perception and esteem [65]. Being eye-to-eye with peers increased inclusion and participation with others, and was frequently raised in qualitative studies in relation to both standing and stepping devices [57,65,71,79,80]. The positive impact on children’s self-esteem, sense of equality and confidence during social interactions was particularly raised in relation to the use of stepping devices along with the ability to move easily between play activities with others [22].

#### 3.1.6. Future

Both standing and stepping positively influence physical health, with opportunities to increase activity and biomechanical loading of the muscle and bone. These may serve to facilitate change in both the form (i.e., volume and quality) and function of the muscle and bone, which in turn may have a greater role in reducing cardiometabolic risk and future health status. Supported standing devices provide more control over body position, alignment and weightbearing [81]; thus, they may be more effective in the maintenance of BMD and contracture prevention, and they can positively impact hip stability. Supported stepping devices promote mobility and may be more effective in promoting muscle development and cardio-respiratory fitness. The impact on psycho-social health of upright positioning and mobility should not be overlooked, due to its positive influence on self-esteem, confidence and the perception of others. Increased communication abilities, and the positive impact on social interaction, attention and learning, may have long-standing influences on children’s future opportunities and achievements.

**Figure 3:**
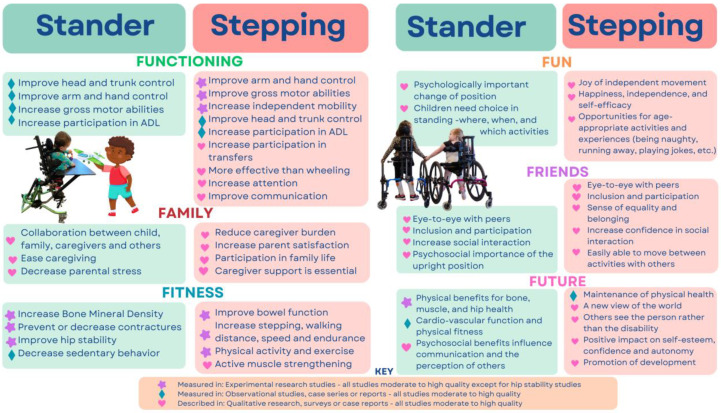
F-words comparison of evidence for supported standing and stepping devices (authors’ original graphic).

Figure 3 compares benefits from using supported standing and supported stepping devices according to the F-words. Evidence is divided into three categories: experimental group studies; observational group or cross-sectional studies, case series or case reports; and qualitative studies, descriptive evidence from surveys, expert opinion or benefits described in case series or reports, but not measured using a valid and reliable outcome measure. Results are reported from the highest quality studies available for each outcome.

### 3.2. iHAAT Conceptual Framework

#### 3.2.1. Human

This domain includes the consideration of the child’s profile, needs, abilities (fitness and functioning), goals and desired roles (friends, fun, family and future). With an interdependence frame in mind, family (parents and other adult caregivers’) goals, desires, typical routines, physical abilities (for transfers) and ability to use more complex equipment should all be considered when choosing between different device types or determining where and when it should be used, as well as what type of device might best meet the goals. In addition, the needs and abilities of other children and adults who will interact with the child while they are using the device should be considered. By enhancing child participation and engagement, supported standing and stepping devices may also increase family, sibling and peer interaction (friends), and there may be a reciprocal impact on the perceptions of peers and the wider community (future) [21,22,65].

#### 3.2.2. Activity

The child’s age, stage and life roles influence the types of activities and participation that may be considered appropriate or fun. Activities are influenced by child abilities (fitness and functioning), child and family goals and preferences (family, fun and future) as well as others who will engage in the activity with the child (friends and family). The interdependence frame would encourage reflection on the reciprocal interactions and impacts on peers, siblings, family and caregivers through engagement in meaningful activities and routines with the child, facilitated by standing and stepping device use.

#### 3.2.3. Assistive Technology

Use of and satisfaction with standing and stepping devices is influenced by everyone who interacts with or assists the child in using that device. Prescription is influenced by the human and activity factors previously discussed, all of which have reciprocal interactions and interdependence. Device prescription is also influenced by the intended goals and both children and family, peers, caregivers and others are interdependent with the AT [42]. 

Fitness and future goals such as maintaining BMD, muscle strength or hip stability may be influenced by weight bearing. Actual weight bearing in standing and stepping devices varies widely [17,82,83,84], and it is influenced by device type (multi-position supine versus sit-to-stand) [83], as well as inclination, hip abduction and orientation (prone, supine or upright) [81]. If impact on BMD or muscle is the desired fitness outcome, then it may be important to measure actual weight bearing to ensure this goal is being met. In contrast, if the desired outcome is inclusion or participation, influencing fun and friends, then other factors such as ease of transfer, child- and family-friendly design, color, transportability, accessories (such as a tray), or the ability to position the child to reach desired activities or interact in chosen routines may be more influential.

#### 3.2.4. Context

The context (physical, social and attitudinal environment) influences and is influenced by the child and their social circle, the assistive device, and the activities and roles desired. The need for caregiver support for success was particularly emphasized in stepping studies [22], while a major theme from the lived-experience supported standing data was that collaboration between the child, family, caregivers and therapist is required for successful standing programs [21].

The physical environment particularly influences the choice of the supported stepping device, since some are only appropriate for smooth indoor surfaces, while others have wheels suitable for use on grass, rough ground outdoors, or may be more effective on carpet or thresholds indoors [85]. Transfers have a major influence on the prescription of both standing and stepping devices, and the space for transfers, the child’s ability to provide assistance, the caregiver’s abilities and the equipment used to facilitate transfers, influence use of these devices [21,22]. 

Parents’ [86,87], educators’ [88] and therapists’ [89,90,91] attitudes all influence prescription and use of standing devices. Children prioritize comfort in a stander, and this may correlate with the therapists’ perceptions of good alignment [90]. While therapists have strong beliefs in the benefits of supported standing for physical health [87], attitudes towards supported stepping devices are mixed. The ‘ON-Time’ mobility framework encourages the use of multiple methods of mobility within the first year of life as a child’s right, but some therapists remain concerned that independence and mobility will be achieved at the cost of ‘normal’ movement patterns [92]. For children with non-ambulant CP, stepping devices should be used to promote inclusion (functioning, fun and friends) and physical activity (Fitness), and these goals should be prioritized over ‘typical’ gait patterns [22]. The reciprocal impact of children’s use of supported stepping devices on the attitudes and perceptions of peers and wider societal attitudes [65] reinforces the interdependence frame. 

#### 3.2.5. Well-Being

Finally, all domains of the iHAAT interact with each other to influence overall quality of life or well-being. For children, well-being is influenced by overall health, happiness, development and functioning at home, at school and out in the community. The F-words approach also emphasizes the importance of a family-centered holistic, strengths-based approach with the overall goal of enhancing well-being [45].

**Figure 4:**
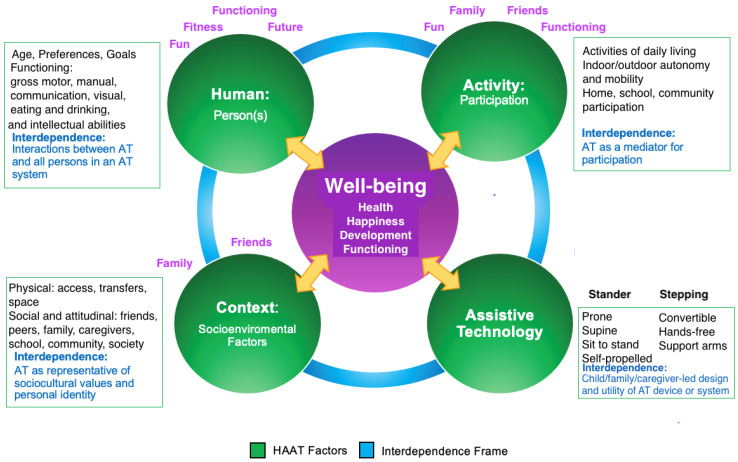
An integration of the iHAAT framework and F-words for children with CP (GMFCS levels IV and V) using supported stepping and supported standing devices. Adapted with permission, copyright 2021, Fani Lee [42].

See Figure 4 for the application of iHAAT to children at GMFCS levels IV or V using supported standing and stepping devices, and relationships between iHAAT components and the F-words. Although AT such as supported standing and stepping devices may help to address all F-words ingredients, the AT is interdependent with the human, activity and context domains. For example, a stander may be used to promote BMD (fitness) within the human domain, or it may be used to promote self-feeding skills (functioning) within the activity domain, or it may be a barrier or facilitator to social participation with peers (friends) within the context domain.

## 4. Discussion

Both standing and stepping devices are well tolerated by children with non-ambulant CP, with an average dosage of 60 min, 5–7 days a week reported for both devices. There is a considerable overlap between the benefits and outcomes of standing and stepping devices, although both increase participation in age-appropriate activities with others. Standers may provide greater benefits for BMD, contracture prevention and hip stability, while stepping devices may provide more benefits for physical fitness, psycho-social, communication and emotional development due to the benefits of self-initiated mobility. 

Around twenty years ago, it was a popular concept that invoking the central pattern generator for stepping would improve gait outcomes in adults with stroke, brain injury or spinal cord injury (SCI). However, large-scale randomized controlled studies failed to support this concept [93]. Today, we understand that reflexes cannot be ‘integrated’ or turned into volitional movements, and the idea of using the central pattern generator for stepping is absent from the contemporary literature. Current clinical practice guidelines for improving walking speed and distance in adults with stroke, brain injury and SCI recommend task-specific overground walking training as being more effective than treadmill or robotic training, particularly for individuals who are at least 6 months post-injury, for the purposes of improving walking speed and distance [93]. 

The most recent (2017) Cochrane review of treadmill training for young children mainly included studies of children with Down syndrome, while only two studies focused on children with CP. A small increase in walking speed was measured for children with developmental delays, and improved motor skills were measured for children at GMFCS levels I or II, but no studies included children at GMFCS levels IV or V [94]. In the CP literature, there has been a shift towards the use of robotic gait devices. However, for children with non-ambulant CP, no significant benefits have been measured, in comparison to children using a combination of treadmill training with overground training in a supported stepping device [95]. An earlier study demonstrated that walking speed and endurance increased in children with CP (GMFCS levels III and IV) who trained overground using walkers and supported stepping devices in comparison to those treadmill training [96]. 

Drawing upon the key intervention ingredients of child-active and task-specific practice [3], intervention approaches seeking to improve walking or stepping for children with CP might opt to incorporate functional overground walking whereby the active use of the plantar flexors and hip and knee extensors is required to move forward. When stepping on a treadmill, there are some limitations as to its ‘child-active’ and ‘task-specific’ ingredients. For example, while the child is required to activate the hip and knee flexors to step forward on a treadmill (unless assisted by an adult), the leg is pulled back passively. Also, while treadmill training might be ideal for fitness benefits, such as increasing heart rate and step count, it does not offer the visual and sensory experience of overground training nor the potential for enhancing participation and engagement with friends. 

Children with non-ambulant CP have increased stepping, achievement of functional goals and improved gross motor skill following intensive locomotor training where they participated in a combination of partial body weight support treadmill training immediately followed by practice overground [95]. This suggests that the ‘warm-up’ on the treadmill may be beneficial for some children as preparation for practice stepping overground. If intensive blocks of treadmill or robotic training are chosen as the family’s preference, they should also be accompanied by overground stepping to promote functioning in real-life contexts and activities. 

It may also be argued that, in comparison to supported stepping devices, power mobility promotes more efficient and effective mobility for children with non-ambulant CP [23,97]. While this is certainly true for larger spaces, such as in school, outdoors and for distance and community mobility, there are limitations with power mobility in smaller homes and early childhood settings [98,99]. There are few developmentally appropriate devices that are sufficiently maneuverable and safe for use in indoor settings with other young children [99,100,101,102]. Children may also have difficulty reaching toys or performing activities from the seated position in a powered wheelchair [103], while children using hands-free stepping devices have increased use of arms and hands as well as engagement in play and participation with others [22,78,104].

In line with the need to decrease sedentary behavior in children with non-ambulant CP, the ON-Time mobility framework would also suggest that multiple modes of mobility should be introduced, starting by 12 months of age. Individuals with non-ambulant CP are reported to spend long periods of time in lying and sitting positions, and they have limited abilities to move without support [105]. Standing at least one hour daily, with an additional hour of supported stepping, may offer enough variability to counter the negative effects of the prolonged time spent in a sitting position. A case report of twins functioning at GMFCS level V demonstrated the feasibility of ON-Time introduction of adaptive seating, standing, stepping and early power mobility devices, with a positive impact on all F-words [106]. This study also reinforced the interdependence between parents, children and siblings, and the impact that assistive device use may have on family functioning and goals.

The use of supported standing, supported stepping and power mobility devices has also been shown to be feasible for a group of young children, 18 months to 7 years, with CP. While not all children had both standing and stepping devices, more than half of the children functioning at GMFCS levels IV or V (19/34) used standing, stepping and power mobility devices concurrently for at least 6 months. A key finding was that the introduction of power mobility did not decrease the use of stepping devices for children at GMFCS levels III, IV or V [107]. Of the 14 children at GMFCS level V who used supported stepping devices, 8/14 maintained and 6/14 increased their time in stepping devices following the introduction of power mobility. Since these children require support to attain or maintain upright positions, parents may have found the variety of complementary positioning and mobility options useful for increasing their functioning and fun. 

Powered ride-on toy studies have demonstrated the feasibility of using these devices in standing positions for infants with Down syndrome [108]. For children with developmental delays, including CP, social skills may be increased by training in a standing position due to an increased visual field of view [109]. However, mobility and social skills have been shown to increase following structured powered ride-on car training in various combinations of sitting and standing positions [110]. A more intense intervention dosage may be required for children with severe disabilities, such as those with non-ambulant CP, and the use of a variety of positioning and mobility devices within meaningful and natural routines may be one way to achieve this.

However, assistive devices are expensive, and this may be a significant barrier with regard to the use of multiple devices in many settings. For the smallest children (in terms of height and weight), typical baby equipment may be used to support standing and stepping in the home and child care environment (e.g., exersaucers or ride-on toys). Bracing, splints or locally sourced materials (paper, plaster, cardboard, wood, etc.) can also be used to provide sufficient time in upright positions as alternatives to more expensive durable medical equipment [111,112,113]. Basic wheeled walkers have also been successfully adapted with low-cost materials to create hands-free supported stepping devices suitable for children with non-ambulant cerebral palsy in lower-resourced settings [62]. 

In the United States (US), the Individuals with Disabilities Education Act (IDEA) ensures free appropriate public education (FAPE) for children with disabilities. This is provided through Individualized Family Service Plans (IFSP) for infants and toddlers as well as Individualized Education Plans (IEP) for school-aged children. Under these programs, assistive technology necessary for FAPE and/or to address IFSP or IEP goals must be provided, maintained, and reassessed yearly; moreover, devices must be available for children to use at home over the weekends and holidays. Therefore, in the US, it may be a matter of the law for supported standing and stepping devices to be provided to address functioning, fitness, fun, friends and future goals for children with mobility limitations. 

In other settings with publicly available funding or loan programs for positioning and mobility devices, the use of multiple positioning and mobility devices may be influenced more by attitudes. Standers or standing frames are often considered first, and stepping devices are not considered until older ages. This hierarchical approach is outdated, and does not consider that children typically pull to stand and begin to step in very close succession. It is known that adults interact differently with children who are standing and stepping, in comparison to those who are lying or sitting [114]. In order to be provided with equitable developmental opportunities, children with non-ambulant CP need multiple ON-Time positioning and mobility opportunities, starting within the first year of life.

### Limitations

This study synthesized the results of scoping reviews conducted by two of the authors, raising questions of possible bias in the selection of citations. However, these reviews were systematically conducted, with protocols and inclusion criteria being registered a priori. They included hand searching and grey literature, in addition to extensive database searches. As with any review, it is possible that unpublished studies, or those conducted in other locations, may have been missed. However, these reviews are the most current and comprehensive evidence sources available in relation to the use of supported standing and stepping interventions for children with non-ambulant CP. 

Evidence level was descriptively summarized in Figure 3 to assist with knowledge translation of scoping review results through the lens of the F-words framework. Results from the highest evidence level and lowest risk of bias/highest quality studies were included where possible. Group experimental, observational and qualitative study evidence was mainly moderate or high quality, other than research on hip stability, which is still limited, as indicated in Section 3. This manuscript is not a scoping or systematic review, and readers are directed to the two original review publications [21,22] for details regarding evidence level, quality and risk of bias analyses.

Two different theoretical frameworks were used in the analysis of this evidence to create an integrated model, which is open to discussion. However, we feel that these complement each other with their different emphases. Although the F-words framework supports a holistic approach to physical activity and rehabilitation interventions, few articles have applied it to interventions for children at GMFCS levels IV or V, and it is not focused on AT. In contrast, the i-HAAT is an AT-specific framework, and it additionally places focus on interdependence, which is critical when considering children with non-ambulant CP. 

## 5. Conclusions

Supported standing and stepping devices should be introduced within the first year of life to promote functioning, family, fitness, fun, friends and future goals as part of ON-Time mobility. The interdependence frame encourages reflection on the reciprocal interactions between the family, caregivers, peers and wider society interacting with children using supported standing and stepping devices to engage and increase their autonomy in meaningful activities and roles within the physical, social and attitudinal environment or context.

This study, and the integrated iHAAT and F-words model, challenge therapists to consider that young children at GMFCS levels IV and V should have opportunities to engage in both supported standing and stepping, in addition to power mobility. Standing and stepping devices afford weightbearing and mobility, that is medically and developmentally necessary. Multiple modes of upright positioning and mobility are needed to provide equitable developmental opportunities for young children with non-ambulant CP.

## Data Availability

The data used in this study are contained within the article.
